# Seroconversion of SARS-CoV-2 Antibodies in Pregnant Women During the Second Wave of the COVID-19 Pandemic in India: A Longitudinal Study

**DOI:** 10.7759/cureus.73677

**Published:** 2024-11-14

**Authors:** Kandala Aparna Sharma, Nilanchali Singh, Anapti Anil, Sara Hillman, Purva Mathur, Kapil Yadav, Shivam Pandey, Vatsla Dadhwal, Saloni Kamboj

**Affiliations:** 1 Department of Obstetrics and Gynecology, All India Institute of Medical Sciences, New Delhi, IND; 2 Department of Obstetrics and Gynecology, University College London, London, GBR; 3 Department of Microbiology, All India Institute of Medical Sciences, New Delhi, IND; 4 Department of Community Medicine, All India Institute of Medical Sciences, New Delhi, IND

**Keywords:** covid antibodies, pandemic, pregnancy, sero-epidemiological, vaccination

## Abstract

Aim

This study aims to investigate the seroconversion of antibodies against SARS-CoV-2 infection in pregnant women during their first and third trimesters, specifically during the second wave of the COVID-19 pandemic.

Methodology

This is a longitudinal follow-up study of pregnant women during the second peak of the COVID-19 pandemic. Patients in their first trimester (six to 12 weeks) were tested for SARS-CoV-2 IgM and IgG levels, with a repeat test conducted in the third trimester (28-40 weeks) using the automated VIDAS® (bioMérieux SA, Marcy-l'Étoile, France) system. Samples were compared to assess seroconversion rates.

Results

In the current study, 120 of the 298 eligible women were followed through to the third trimester. Seroconversion of SARS-CoV-2 IgG from the first to third trimester was observed in 28 women (23.3%), including six women (21.4%) who developed SARS-CoV-2 IgG antibodies in the third trimester, and 22 women (78.5%) who were IgG positive in the first trimester but became IgG negative by the third trimester. Seroconversion of SARS-CoV-2 IgM from the first to third trimester was observed in 15 women (12.5%), of whom three women (25%) developed SARS-CoV-2 IgM antibodies in the third trimester, while 12 women (80%) who had these antibodies earlier no longer retained them by the third trimester.

Conclusions

The seroconversion rate during pregnancy is high, and natural immunity acquired through infection may not provide long-term protection, even over the course of the pregnancy.

## Introduction

The understanding of COVID-19, including its defining characteristics and infectivity, has improved significantly over the past two years. However, the immunological response to COVID-19 during pregnancy remains less well-defined. The WHO’s global research map for COVID-19 recommends population-level seroepidemiological studies to generate data on infection levels and inform containment strategies [1]. We previously published data on the seroprevalence of SARS-CoV-2 antibodies among first-trimester pregnant women during the second wave of the pandemic in India [2]. Seroepidemiology is a valuable tool for designing and monitoring vaccination programs [3-5]. In the context of pregnancy, seroepidemiological studies can guide preventive strategies, including vaccination planning. This study aims to investigate the seroconversion of antibodies against SARS-CoV-2 in pregnant women during their first and third trimesters of the pandemic, with findings that could inform policy decisions regarding vaccination and potential fetal transmission.

## Materials and methods

This was a longitudinal observational study of pregnant women who had not been formally diagnosed with COVID-19 in the past three months, visiting the rural center of All India Institute of Medical Sciences, New Delhi, New Delhi, India, during the second peak of the COVID-19 pandemic, from April 2021 to August 2021. Inclusion criteria were (1) low-risk pregnant women who had not been diagnosed with COVID-19 infection in the past three months; (2) participants willing to follow up; and (3) participants willing to get tested twice during the study period for SARS-CoV-2 IgG and IgM antibodies. Exclusion criteria were (1) pregnant women diagnosed with COVID-19 infection in the past three months; (2) multiple pregnancies; (3) participants who refused to follow up or get tested during the study period; and (4) women who received the COVID-19 vaccine.

Data on demographic characteristics and COVID-19-related symptoms were collected using a structured questionnaire by a professional healthcare worker. Patients in the first trimester (six to 12 weeks) were tested for SARS-CoV-2 IgM and IgG levels, and a repeat test for the same was done in the third trimester (28-40 weeks). VIDAS® (bioMérieux SA, Marcy-l'Étoile, France) SARS-CoV-2 IgM (qualitative) and VIDAS® SARS-CoV-2 IgG II (semi-quantitative) assays were used with the automated VIDAS® system to detect IgM and IgG, respectively, in both the first and third trimesters.

Samples from the first trimester were compared to those collected in the third trimester from the same patient to interrogate seroconversion rates. Ethical approval was obtained from the institutional ethical committee via approval no. IEC-38/08.01.2021, stating “Approved from ethical angle prospectively w.e.f 27th Jan 2021.” Written informed consent was obtained from all participants.

Data analysis was carried out using Stata version 16.0 (StataCorp LLC, College Station, Texas, USA). Quantitative variables were expressed as the mean ± SD, and qualitative categorical variables were expressed as frequency and percentages. Mean values of normally distributed data were compared using the Student’s t-test. Qualitative variables were compared using the χ² test or Fisher’s exact test, as appropriate.

## Results

In the current study, of the 298 eligible women, 120 were followed through to the third trimester. Participants’ gestational age at booking ranged from six to 20 weeks. The average age of the participants was 24 years, and the average BMI was 22.52 kg/m². Most participants were homemakers, and none were smokers. Additionally, none had comorbidities such as autoimmune disease, essential hypertension, chronic kidney disease, or type 1 or type 2 diabetes, with the exception of one participant who had hypertension. None had a family history of diabetes mellitus or hypertension. The study population was considered low-risk because the rural center primarily caters to low-risk patients, with high-risk patients being referred to higher-level centers. Demographic details are summarized in Table 1.

**Table 1:** Demographic data of the study population

Characteristics	IgG positive (n = 72)	IgG negative (n = 48)	p-value	IgM positive (n = 14)	IgM negative (n = 106)	p-value
Mean age in years	24.26	23.47	0.3254	23.35	24.02	0.5821
Mean gestational age at booking	14.1	13.5	0.4485	13.5	14	0.8012
Mean BMI (kg/m²)	22.86	22.36	0.5121	25	22.21	0.9
Multiparity	15	9	0.579	2	22	0.9

None of the women initially presented with symptoms; however, all were questioned about possible signs of infection in the past month. This revealed that 12 women (10%) reported fever, six (5%) had a cough, three (2.5%) experienced shortness of breath, three (2.5%) had a sore throat, three (2.5%) had fever with cough, one (0.83%) had fever with shortness of breath, and one (0.83%) had fever with headache. None of the women experienced lethargy, vomiting, joint pain, diarrhea, rhinorrhea, loss of taste or smell, or any other symptoms. Nasopharyngeal and throat swabs for COVID-19 RT-PCR were negative for all participants. None were aware of any exposure to a COVID-19 case at home, in the community, or in the hospital, and none had a history of traveling abroad. The prevalence of antibodies in relation to symptomatology among the participants is shown in Table 2 and Table 3. No significant correlation was found between the presence or absence of symptoms and antibody status.

**Table 2:** Prevalence of IgG antibodies in relation to symptomatology among the participants

Status of symptoms	IgG positive (%)	IgG negative (%)	Exact significance (two-sided)
Symptoms present	11 (15.3%)	0 (0%)	0.9
Symptoms absent	61 (84.7%)	48 (100%)	
Total	72 (100%)	48 (100%)	

**Table 3:** Prevalence of IgM antibodies in relation to symptomatology among the participants

Status of symptoms	IgM positive (%)	IgM negative (%)	Exact significance (two-sided)
Symptoms present	1 (7.1%)	17 (16%)	0.691
Symptoms absent	13 (92.9%)	89 (84%)	
Total	14 (100%)	106 (100%)	

Twenty-one women (17.5%) did not have either IgG or IgM antibodies, indicating they were at high risk of developing a COVID-19 infection. Seroconversion from the first to third trimester was observed in 28 women (23.3%). Of these, six women (21.4%) developed SARS-CoV-2 IgG antibodies in the third trimester, while 22 women (78.5%) were IgG positive in the first trimester but became IgG negative in the third trimester (Table 4).

**Table 4:** Seroconversion of IgG antibodies from first trimester to third trimester

	IgG negative in the third trimester	IgG positive in the third trimester	Total
IgG negative in the first trimester	26 (81.25%)	6 (18.75%)	32 (100%)
IgG positive in the first trimester	22 (25%)	66 (75%)	88 (100%)
Total	48 (40%)	72 (60%)	120 (100%)

Seroconversion of SARS-CoV-2 IgM from the first to the third trimester was observed in 15 women (12.5%). Of these, three women (25%) developed SARS-CoV-2 IgM antibodies in the third trimester, while 12 women (80%) who had IgM antibodies in the first trimester no longer had them upon follow-up (Table 5).

**Table 5:** Seroconversion of IgM antibodies from first trimester to third trimester

	IgM negative in the third trimester	IgM positive in the third trimester	Total
IgM negative in the first trimester	94 (96.91%)	3 (3.09%)	97 (100%)
IgM positive in the first trimester	12 (52.17%)	11 (47.83%)	23 (100%)
Total	106 (88.33%)	14 (11.67%)	120 (100%)

However, among the 11 women (9.1%) who remained IgM positive in the third trimester, six women (54.5%) also tested IgG positive.

## Discussion

Our previous study of 220 pregnant women demonstrated the seroprevalence of SARS-CoV-2 antibodies in the first trimester. Of the participants, 72.7% (n = 160) were SARS-CoV-2 IgG positive, 16.8% (n = 37) were SARS-CoV-2 IgM positive, and 16.9% (n = 27) were positive for both IgG and IgM. The average antibody titers found were 10.49 BAU/ml (±14.0) for anti-SARS-CoV-2 IgG and 0.6 (±0.55) for anti-SARS-CoV-2 IgM non-neutralizing antibodies. ROC analysis for SARS-CoV-2 IgG positivity showed a cutoff value of 1.19, with a sensitivity of 99.3% (AUC 0.99, 95% CI) and specificity of 98.3% (AUC 0.99, 95% CI). Similarly, ROC analysis for SARS-CoV-2 IgM positivity showed a cutoff value of 1, with a sensitivity of 97.3% (AUC 0.99, 95% CI) and specificity of 98.9% (AUC 0.99, 95% CI) [2].

In the present study, approximately 15% of pregnant women seroconverted from negative to positive antibody status against SARS-CoV-2 between the first and third trimesters. This suggests that pregnant women may acquire SARS-CoV-2 antibodies during pregnancy, particularly in the third trimester. The seroconversion rates observed in our study are consistent with those reported by Crovetto et al., who found that approximately 15-20% of pregnant women exhibited seroconversion between the first and third trimesters [6]. Additionally, a study by Zhao et al. reported even higher seroconversion rates, especially among women with moderate to severe COVID-19 symptoms, where antibody production was both stronger and more persistent. This highlights that seroconversion rates and antibody levels may vary depending on disease severity [7].

Implications for vaccination policy

Our study’s finding that 78.5% of women who were IgG-positive in the first trimester became IgG-negative by the third trimester raises concerns about the durability of natural immunity to SARS-CoV-2. This suggests that the natural immune response may not provide long-term protection during pregnancy, emphasizing that reliance on natural infection alone is insufficient for safeguarding against the virus. In contrast, vaccination has been shown to elicit a robust immune response in pregnant women, and recent studies indicate that vaccines can confer passive immunity to the fetus [8]. The timing of vaccination is also crucial, with evidence suggesting that third-trimester vaccination optimizes the transfer of protective antibodies to the fetus.

Future research directions

Our study underscores the necessity for further research into the kinetics of immune responses in pregnant women, especially regarding vaccination. Future investigations could focus on determining the optimal timing for COVID-19 vaccination during pregnancy to enhance fetal protection, as well as assessing the long-term outcomes for both mothers and infants. Additionally, exploring the role of booster doses during pregnancy would be valuable, considering the observed decline in antibody levels over time.

Limitations of the study

The outcomes of SARS-CoV-2 antibodies in both the mother and fetus were not examined in the present study. Additionally, the study experienced an increased number of dropouts due to the pandemic conditions, resulting in a smaller sample size.

## Conclusions

Observing seroprevalence in a vulnerable population is an essential tool for combating disease, managing resources, and communicating public health messages. This longitudinal observational study investigating the seroconversion of SARS-CoV-2 antibodies in pregnant women during the second COVID-19 pandemic provides valuable insights into the evolution of natural immunity throughout pregnancy. Our findings show that while a significant proportion of women seroconvert during pregnancy - approximately 15% exhibit IgG and IgM seroconversion from the first to the third trimester - many women who were initially IgG-positive in the first trimester lose detectable antibody levels by the third trimester. This suggests that natural immunity to SARS-CoV-2 may be short-lived in pregnant women.

The American College of Obstetricians and Gynecologists strongly recommends COVID-19 vaccination for pregnant individuals, as the risk of severe illness and death is elevated during pregnancy. Therefore, a key takeaway from our study is the importance of vaccination during pregnancy, not only to enhance and maintain a stable immune response but also to provide the potential for passive immunity transfer to the fetus.

## Appendices

Proforma

Hospital Number:         Patient Code:    Name:      Age:     Gestational Age at Booking:     

Pregnancy details         Parity:                          Previous Delivery Details:       

BMI:    Ethnic Background:     

Patient Occupation:      Partner Occupation:      Smoker:

Medical History:

Autoimmune Disease:               Essential Hypertension:            Diabetes Mellitus:       

Chronic Kidney Disease:                      Assisted Reproduction:             Multiple Pregnancy:

Pregnancy Interval > 10 years:

Family History:            

Family History of PET  Family History of DM (1st degree relative)

Previous Pregnancy Complication

Previous Pregnancy induced Hypertension/ PET

Previous GDM

Previous stillbirth

Previous IUGR

Previous baby 4.5kg or above

First trimester visit

Blood Group:    Hemogram:       HIV:     HbsAg:             VDRL:              TSH:       HBA1C:           Urine microscopy:

COVID-19 Negative/ Positive:

Blood test for COVID antibody:            IgM Titres:        IgG Titres:

Symptoms of COVID-19 in 1st Trimester:

Fever   Vomiting          Cough              Diarrhoea         Rhinorrhoea    Sore throat       Headache         Lethargy          Joint pain         Loss of taste / smell       Other symptoms           Exposure in community            Exposure in hospital     Travel abroad

Needing assessment in Bacterial infection including chest         Venous Thromboembolism (PE/DVT)   Need for oxygen  Needing admission to hospital   need for intubation        Admission to ICU

COVID-19 Treatment in 1st Trimester:                                                     COVID-19 Vaccination given: yes/no

Investigations in Current Pregnancy

NTNB Scan:                 Level II Scan:

Other Investigations:

Third trimester

Hemogram:

COVID-19 Negative/ Positive:

Blood test for COVID antibody:            IgM titres:         IgG titres:

Symptoms of COVID-19 in 2nd Trimester:

Fever   Vomiting          Cough              Diarrhoea         Rhinorrhoea    Sore throat       Headache         Lethargy          Joint pain         Loss of taste / smell         Other symptoms           Exposure in community            Exposure in hospital     Travel abroad

Needing assessment in  Bacterial infection including chest         Venous Thromboembolism (PE/DVT)   Need for oxygen

Needing admission to hospital   need for intubation        Admission to ICU

COVID-19 Treatment in 2nd Trimester:                                                         COVID-19 Vaccination given: yes/no
